# Supplementary material for: A self-balancing circuit centered on MoOsm1 kinase governs adaptive responses to host-derived ROS in Magnaporthe oryzae
Source: eLife. 2020 Dec 4;9:e61605. doi: 10.7554/eLife.61605 (PMC7717906; doi:10.7554/eLife.61605)
Supplement: Supplementary file 1. — Genomic location and Annotation ID of MoAtf1 binding genes. [file elife-61605-supp1.docx]

**Table S1. 574 putative binding proteins of MoAtf1 by CHIP assay.**

1 chr1 884383 884785 ChIP_peak_3 MGG_13736

2 chr1 884383 884785 ChIP_peak_3 MGG_02186

3 chr1 1167442 1168238 ChIP_peak_5 MGG_02256

4 chr1 1167442 1168238 ChIP_peak_5 MGG_02257

5 chr1 1483976 1484393 ChIP_peak_7 MGG_02346

6 chr1 1483976 1484393 ChIP_peak_7 MGG_02347

7 chr1 2254635 2255141 ChIP_peak_10 MGG_16144

8 chr1 2254635 2255141 ChIP_peak_10 MGG_11882

9 chr1 2398704 2399011 ChIP_peak_11 MGG_11876

10 chr1 2416031 2416340 ChIP_peak_12 MGG_06694

11 chr1 2416031 2416340 ChIP_peak_12 MGG_06695

12 chr1 2427117 2427500 ChIP_peak_13 MGG_06696

13 chr1 2602132 2602446 ChIP_peak_14 MGG_06751

14 chr1 2602132 2602446 ChIP_peak_14 MGG_06752

15 chr1 2609351 2609841 ChIP_peak_15 MGG_06753

16 chr1 2609351 2609841 ChIP_peak_15 MGG_16162

17 chr1 2609351 2609841 ChIP_peak_15 MGG_06755

18 chr1 2672440 2672929 ChIP_peak_16 MGG_06769

19 chr1 2676037 2676449 ChIP_peak_17 MGG_06770

20 chr1 2723774 2724466 ChIP_peak_18 MGG_06781

21 chr1 2723774 2724466 ChIP_peak_18 MGG_06782

22 chr1 2723774 2724466 ChIP_peak_18 MGG_06780

23 chr1 3369389 3370003 ChIP_peak_20 MGG_06955

24 chr1 3369389 3370003 ChIP_peak_20 MGG_06956

25 chr1 3369389 3370003 ChIP_peak_20 MGG_06957

26 chr1 3552281 3552852 ChIP_peak_21 MGG_16238

27 chr1 3552281 3552852 ChIP_peak_21 MGG_16239

28 chr1 3568713 3569014 ChIP_peak_22 MGG_07009

29 chr1 3568713 3569014 ChIP_peak_22 MGG_07010

30 chr1 3568713 3569014 ChIP_peak_22 MGG_07011

31 chr1 3965310 3966360 ChIP_peak_23 MGG_09260

32 chr1 3965310 3966360 ChIP_peak_23 MGG_09259

33 chr1 4056615 4057121 ChIP_peak_25 MGG_11739

34 chr1 4056615 4057121 ChIP_peak_25 MGG_16265

35 chr1 4108843 4109699 ChIP_peak_26 MGG_11731

36 chr1 4108843 4109699 ChIP_peak_26 MGG_14629

37 chr1 4242613 4243055 ChIP_peak_28 MGG_09891

38 chr1 4242613 4243055 ChIP_peak_28 MGG_11727

39 chr1 4454994 4455430 ChIP_peak_30 MGG_09949

40 chr1 4454994 4455430 ChIP_peak_30 MGG_16302

41 chr1 4463237 4463705 ChIP_peak_31 MGG_11715

42 chr1 4463237 4463705 ChIP_peak_31 MGG_14547

43 chr1 4463237 4463705 ChIP_peak_31 MGG_11713

44 chr1 4490398 4490998 ChIP_peak_32 MGG_09955

45 chr1 4490398 4490998 ChIP_peak_32 MGG_09954

46 chr1 4492003 4493558 ChIP_peak_33 MGG_09955

47 chr1 4492003 4493558 ChIP_peak_33 MGG_09956

48 chr1 4492003 4493558 ChIP_peak_33 MGG_09954

49 chr1 4536059 4536491 ChIP_peak_34 MGG_10650

50 chr1 4536059 4536491 ChIP_peak_34 MGG_11710

51 chr1 4627340 4628915 ChIP_peak_35 MGG_15143

52 chr1 4680819 4681452 ChIP_peak_36 MGG_10514

53 chr1 4680819 4681452 ChIP_peak_36 MGG_10515

54 chr1 4715206 4715723 ChIP_peak_37 MGG_10525

55 chr1 4715206 4715723 ChIP_peak_37 MGG_10526

56 chr1 5185512 5185813 ChIP_peak_38 MGG_05427

57 chr1 5185512 5185813 ChIP_peak_38 MGG_05428

58 chr1 5516769 5517164 ChIP_peak_41 MGG_16392

59 chr1 5516769 5517164 ChIP_peak_41 MGG_16393

60 chr1 5516769 5517164 ChIP_peak_41 MGG_05528

61 chr1 5537830 5538269 ChIP_peak_42 MGG_16394

62 chr1 5537830 5538269 ChIP_peak_42 MGG_15176

63 chr1 5537830 5538269 ChIP_peak_42 MGG_13107

64 chr1 5537830 5538269 ChIP_peak_42 MGG_15175

65 chr1 5540968 5541393 ChIP_peak_43 MGG_15176

66 chr1 5540968 5541393 ChIP_peak_43 MGG_13107

67 chr1 5540968 5541393 ChIP_peak_43 MGG_16395

68 chr1 5691704 5692181 ChIP_peak_44 MGG_05572

69 chr1 5691704 5692181 ChIP_peak_44 MGG_16409

70 chr1 5691704 5692181 ChIP_peak_44 MGG_16410

71 chr1 5719909 5720249 ChIP_peak_45 MGG_13121

72 chr1 5719909 5720249 ChIP_peak_45 MGG_05584

73 chr1 5921131 5921519 ChIP_peak_46 MGG_16428

74 chr1 5921131 5921519 ChIP_peak_46 MGG_16429

75 chr1 5921131 5921519 ChIP_peak_46 MGG_05636

76 chr1 6104563 6104941 ChIP_peak_47 MGG_05682

77 chr1 6104563 6104941 ChIP_peak_47 MGG_05683

78 chr1 6496462 6496799 ChIP_peak_50 MGG_16472

79 chr1 6496462 6496799 ChIP_peak_50 MGG_10607

80 chr1 6579647 6580053 ChIP_peak_52 MGG_16477

81 chr1 6579647 6580053 ChIP_peak_52 MGG_10634

82 chr1 6579647 6580053 ChIP_peak_52 MGG_14299

83 chr1 6854279 6854853 ChIP_peak_53 MGG_16504

84 chr1 6854279 6854853 ChIP_peak_53 MGG_04727

85 chr1 7196183 7196583 ChIP_peak_55 MGG_04562

86 chr1 7196183 7196583 ChIP_peak_55 MGG_04561

87 chr1 7196183 7196583 ChIP_peak_55 MGG_04560

88 chr2 63103 63535 ChIP_peak_57 MGG_15494

89 chr2 63103 63535 ChIP_peak_57 MGG_10993

90 chr2 83670 84040 ChIP_peak_58 MGG_10744

91 chr2 83670 84040 ChIP_peak_58 MGG_10745

92 chr2 569628 570002 ChIP_peak_59 MGG_10957

93 chr2 569628 570002 ChIP_peak_59 MGG_14735

94 chr2 948956 949445 ChIP_peak_60 MGG_04534

95 chr2 948956 949445 ChIP_peak_60 MGG_04536

96 chr2 1267242 1267583 ChIP_peak_61 MGG_15557

97 chr2 1267242 1267583 ChIP_peak_61 MGG_15558

98 chr2 1269574 1269967 ChIP_peak_62 MGG_15558

99 chr2 1269574 1269967 ChIP_peak_62 MGG_15559

100 chr2 1395665 1396441 ChIP_peak_65 MGG_04764

101 chr2 1395665 1396441 ChIP_peak_65 MGG_04768

102 chr2 1395665 1396441 ChIP_peak_65 MGG_04769

103 chr2 1774839 1775224 ChIP_peak_70 MGG_08825

104 chr2 1774839 1775224 ChIP_peak_70 MGG_08826

105 chr2 1892589 1892942 ChIP_peak_71 MGG_08856

106 chr2 1892589 1892942 ChIP_peak_71 MGG_08857

107 chr2 1976711 1977379 ChIP_peak_73 MGG_14759

108 chr2 1976711 1977379 ChIP_peak_73 MGG_08883

109 chr2 2113296 2113878 ChIP_peak_75 MGG_08918

110 chr2 2113296 2113878 ChIP_peak_75 MGG_08919

111 chr2 2130357 2130850 ChIP_peak_76 MGG_15610

112 chr2 2130357 2130850 ChIP_peak_76 MGG_08922

113 chr2 2516832 2517279 ChIP_peak_77 MGG_01174

114 chr2 2516832 2517279 ChIP_peak_77 MGG_01175

115 chr2 2614962 2615321 ChIP_peak_79 MGG_01198

116 chr2 2614962 2615321 ChIP_peak_79 MGG_11271

117 chr2 2663312 2663769 ChIP_peak_80 MGG_01207

118 chr2 2663312 2663769 ChIP_peak_80 MGG_01208

119 chr2 2663312 2663769 ChIP_peak_80 MGG_01209

120 chr2 2848686 2849018 ChIP_peak_81 MGG_14773

121 chr2 2848686 2849018 ChIP_peak_81 MGG_15654

122 chr2 2848686 2849018 ChIP_peak_81 MGG_15655

123 chr2 2857924 2858351 ChIP_peak_82 MGG_14526

124 chr2 2857924 2858351 ChIP_peak_82 MGG_14774

125 chr2 2962215 2962640 ChIP_peak_83 MGG_11239

126 chr2 2962215 2962640 ChIP_peak_83 MGG_01282

127 chr2 2962215 2962640 ChIP_peak_83 MGG_01284

128 chr2 3177180 3177645 ChIP_peak_85 MGG_01341

129 chr2 3177180 3177645 ChIP_peak_85 MGG_01342

130 chr2 3177180 3177645 ChIP_peak_85 MGG_15673

131 chr2 3177909 3178348 ChIP_peak_86 MGG_01341

132 chr2 3177909 3178348 ChIP_peak_86 MGG_01342

133 chr2 3177909 3178348 ChIP_peak_86 MGG_15673

134 chr2 3299298 3299643 ChIP_peak_87 MGG_01373

135 chr2 3299298 3299643 ChIP_peak_87 MGG_01374

136 chr2 3309980 3310615 ChIP_peak_88 MGG_15684

137 chr2 3309980 3310615 ChIP_peak_88 MGG_01376

138 chr2 3629436 3629870 ChIP_peak_91 MGG_01463

139 chr2 3629436 3629870 ChIP_peak_91 MGG_15704

140 chr2 3721388 3721823 ChIP_peak_92 MGG_01479

141 chr2 3721388 3721823 ChIP_peak_92 MGG_01480

142 chr2 3846487 3846840 ChIP_peak_93 MGG_01503

143 chr2 3846487 3846840 ChIP_peak_93 MGG_01504

144 chr2 3846487 3846840 ChIP_peak_93 MGG_01505

145 chr2 3885485 3885880 ChIP_peak_95 MGG_15726

146 chr2 3885485 3885880 ChIP_peak_95 MGG_01519

147 chr2 3885485 3885880 ChIP_peak_95 MGG_01518

148 chr2 3982590 3982922 ChIP_peak_96 MGG_01551

149 chr2 3982590 3982922 ChIP_peak_96 MGG_01550

150 chr2 4098149 4098534 ChIP_peak_98 MGG_01585

151 chr2 4098149 4098534 ChIP_peak_98 MGG_01586

152 chr2 4098149 4098534 ChIP_peak_98 MGG_01584

153 chr2 4973021 4975326 ChIP_peak_100 MGG_15777

154 chr2 4973021 4975326 ChIP_peak_100 MGG_15778

155 chr2 4973021 4975326 ChIP_peak_100 MGG_15779

156 chr2 4973021 4975326 ChIP_peak_100 MGG_01822

157 chr2 4992525 4993020 ChIP_peak_102 MGG_01826

158 chr2 4992525 4993020 ChIP_peak_102 MGG_11130

159 chr2 5036131 5036670 ChIP_peak_103 MGG_01834

160 chr2 5036131 5036670 ChIP_peak_103 MGG_01835

161 chr2 5440408 5440766 ChIP_peak_105 MGG_08021

162 chr2 5440408 5440766 ChIP_peak_105 MGG_08020

163 chr2 5461460 5462003 ChIP_peak_106 MGG_08026

164 chr2 5547602 5548315 ChIP_peak_108 MGG_08054

165 chr2 5547602 5548315 ChIP_peak_108 MGG_08055

166 chr2 5887981 5888402 ChIP_peak_109 MGG_08156

167 chr2 5887981 5888402 ChIP_peak_109 MGG_12302

168 chr2 5968682 5969297 ChIP_peak_110 MGG_15834

169 chr2 5968682 5969297 ChIP_peak_110 MGG_12308

170 chr2 5968682 5969297 ChIP_peak_110 MGG_12307

171 chr2 6095808 6096264 ChIP_peak_112 MGG_08212

172 chr2 6242107 6242467 ChIP_peak_113 MGG_15846

173 chr2 6242107 6242467 ChIP_peak_113 MGG_07065

174 chr2 6263626 6263943 ChIP_peak_114 MGG_15074

175 chr2 6263626 6263943 ChIP_peak_114 MGG_15075

176 chr2 6263626 6263943 ChIP_peak_114 MGG_12329

177 chr2 6263626 6263943 ChIP_peak_114 MGG_12330

178 chr2 6583174 6583541 ChIP_peak_115 MGG_07157

179 chr2 6583174 6583541 ChIP_peak_115 MGG_07158

180 chr2 6631221 6631762 ChIP_peak_116 MGG_07171

181 chr2 6631221 6631762 ChIP_peak_116 MGG_15869

182 chr2 6631221 6631762 ChIP_peak_116 MGG_12366

183 chr2 6763172 6763755 ChIP_peak_118 MGG_07205

184 chr2 6763172 6763755 ChIP_peak_118 MGG_07206

185 chr2 6785555 6786362 ChIP_peak_119 MGG_07211

186 chr2 6785555 6786362 ChIP_peak_119 MGG_07212

187 chr2 6785555 6786362 ChIP_peak_119 MGG_07213

188 chr2 6910337 6910770 ChIP_peak_121 MGG_07235

189 chr2 6910337 6910770 ChIP_peak_121 MGG_15884

190 chr2 6910337 6910770 ChIP_peak_121 MGG_07237

191 chr2 6910337 6910770 ChIP_peak_121 MGG_07238

192 chr2 7121547 7121930 ChIP_peak_123 MGG_07289

193 chr2 7121547 7121930 ChIP_peak_123 MGG_07290

194 chr2 7121547 7121930 ChIP_peak_123 MGG_07291

195 chr2 7323737 7324185 ChIP_peak_124 MGG_07341

196 chr2 7323737 7324185 ChIP_peak_124 MGG_15090

197 chr2 7323737 7324185 ChIP_peak_124 MGG_07340

198 chr2 7404956 7405389 ChIP_peak_125 MGG_07366

199 chr2 7404956 7405389 ChIP_peak_125 MGG_07368

200 chr2 7404956 7405389 ChIP_peak_125 MGG_07367

201 chr2 7410538 7411152 ChIP_peak_126 MGG_07369

202 chr2 7799867 7800200 ChIP_peak_127 MGG_12466

203 chr2 7799867 7800200 ChIP_peak_127 MGG_08331

204 chr2 7799867 7800200 ChIP_peak_127 MGG_08332

205 chr2 7814504 7815077 ChIP_peak_128 MGG_08325

206 chr2 7814504 7815077 ChIP_peak_128 MGG_08326

207 chr2 8089785 8090204 ChIP_peak_131 MGG_08258

208 chr2 8089785 8090204 ChIP_peak_131 MGG_08257

209 chr2 8093400 8093808 ChIP_peak_132 MGG_08257

210 chr2 8093400 8093808 ChIP_peak_132 MGG_08256

211 chr3 233588 233892 ChIP_peak_133 MGG_16594

212 chr3 233588 233892 ChIP_peak_133 MGG_16595

213 chr3 236612 237328 ChIP_peak_134 MGG_16595

214 chr3 236612 237328 ChIP_peak_134 MGG_16596

215 chr3 242820 243140 ChIP_peak_135 MGG_09117

216 chr3 242820 243140 ChIP_peak_135 MGG_09118

217 chr3 753149 753455 ChIP_peak_138 MGG_07470

218 chr3 753149 753455 ChIP_peak_138 MGG_07472

219 chr3 753149 753455 ChIP_peak_138 MGG_07471

220 chr3 767273 767747 ChIP_peak_139 MGG_07475

221 chr3 767273 767747 ChIP_peak_139 MGG_07476

222 chr3 790681 791181 ChIP_peak_140 MGG_07482

223 chr3 790681 791181 ChIP_peak_140 MGG_15205

224 chr3 790681 791181 ChIP_peak_140 MGG_16646

225 chr3 1159022 1159342 ChIP_peak_142 MGG_07598

226 chr3 1159022 1159342 ChIP_peak_142 MGG_07599

227 chr3 1159022 1159342 ChIP_peak_142 MGG_07600

228 chr3 1165303 1165769 ChIP_peak_143 MGG_13428

229 chr3 1165303 1165769 ChIP_peak_143 MGG_13429

230 chr3 1363043 1363354 ChIP_peak_144 MGG_14523

231 chr3 1401321 1401946 ChIP_peak_145 MGG_07660

232 chr3 1401321 1401946 ChIP_peak_145 MGG_16678

233 chr3 1818899 1819200 ChIP_peak_146 MGG_05881

234 chr3 1818899 1819200 ChIP_peak_146 MGG_05880

235 chr3 1863576 1863963 ChIP_peak_147 MGG_05897

236 chr3 1863576 1863963 ChIP_peak_147 MGG_13279

237 chr3 2215779 2216408 ChIP_peak_150 MGG_05992

238 chr3 2215779 2216408 ChIP_peak_150 MGG_05993

239 chr3 2247378 2247882 ChIP_peak_151 MGG_05999

240 chr3 2247378 2247882 ChIP_peak_151 MGG_06000

241 chr3 2247378 2247882 ChIP_peak_151 MGG_06001

242 chr3 2271986 2272385 ChIP_peak_152 MGG_06010

243 chr3 2271986 2272385 ChIP_peak_152 MGG_06011

244 chr3 2401966 2402310 ChIP_peak_154 MGG_06052

245 chr3 2401966 2402310 ChIP_peak_154 MGG_06053

246 chr3 2681270 2681696 ChIP_peak_156 MGG_06133

247 chr3 2681270 2681696 ChIP_peak_156 MGG_06134

248 chr3 2876161 2876489 ChIP_peak_158 MGG_06190

249 chr3 2876161 2876489 ChIP_peak_158 MGG_06191

250 chr3 2922112 2922481 ChIP_peak_159 MGG_06200

251 chr3 2922112 2922481 ChIP_peak_159 MGG_06201

252 chr3 2922112 2922481 ChIP_peak_159 MGG_06202

253 chr3 3032585 3033274 ChIP_peak_160 MGG_05805

254 chr3 3032585 3033274 ChIP_peak_160 MGG_05804

255 chr3 3558086 3558878 ChIP_peak_162 MGG_09474

256 chr3 3558086 3558878 ChIP_peak_162 MGG_09473

257 chr3 3558086 3558878 ChIP_peak_162 MGG_12989

258 chr3 3558086 3558878 ChIP_peak_162 MGG_16805

259 chr3 3558086 3558878 ChIP_peak_162 MGG_16804

260 chr3 3668595 3668993 ChIP_peak_163 MGG_10912

261 chr3 4041865 4042275 ChIP_peak_167 MGG_04946

262 chr3 4041865 4042275 ChIP_peak_167 MGG_04947

263 chr3 4041865 4042275 ChIP_peak_167 MGG_04948

264 chr3 4045291 4045665 ChIP_peak_168 MGG_04947

265 chr3 4045291 4045665 ChIP_peak_168 MGG_04948

266 chr3 4045291 4045665 ChIP_peak_168 MGG_04949

267 chr3 4055583 4055958 ChIP_peak_169 MGG_16835

268 chr3 4055583 4055958 ChIP_peak_169 MGG_16836

269 chr3 4055583 4055958 ChIP_peak_169 MGG_15119

270 chr3 4055583 4055958 ChIP_peak_169 MGG_04953

271 chr3 4055583 4055958 ChIP_peak_169 MGG_04954

272 chr3 4383373 4384327 ChIP_peak_170 MGG_05039

273 chr3 4383373 4384327 ChIP_peak_170 MGG_05040

274 chr3 4413222 4413777 ChIP_peak_171 MGG_05047

275 chr3 4413222 4413777 ChIP_peak_171 MGG_16850

276 chr3 4413222 4413777 ChIP_peak_171 MGG_16851

277 chr3 4463509 4463949 ChIP_peak_172 MGG_05061

278 chr3 4463509 4463949 ChIP_peak_172 MGG_05062

279 chr3 4463509 4463949 ChIP_peak_172 MGG_05063

280 chr3 4465684 4466093 ChIP_peak_173 MGG_05062

281 chr3 4465684 4466093 ChIP_peak_173 MGG_05063

282 chr3 4465684 4466093 ChIP_peak_173 MGG_05064

283 chr3 4512797 4513269 ChIP_peak_174 MGG_05074

284 chr3 4655795 4656325 ChIP_peak_175 MGG_05116

285 chr3 4655795 4656325 ChIP_peak_175 MGG_16873

286 chr3 4655795 4656325 ChIP_peak_175 MGG_05118

287 chr3 4715443 4715783 ChIP_peak_176 MGG_12936

288 chr3 4715443 4715783 ChIP_peak_176 MGG_05134

289 chr3 4791312 4791839 ChIP_peak_178 MGG_16877

290 chr3 4791312 4791839 ChIP_peak_178 MGG_05158

291 chr3 4791312 4791839 ChIP_peak_178 MGG_05159

292 chr3 4791312 4791839 ChIP_peak_178 MGG_05160

293 chr3 4809388 4810204 ChIP_peak_179 MGG_12927

294 chr3 4809388 4810204 ChIP_peak_179 MGG_05164

295 chr3 5068857 5069357 ChIP_peak_180 MGG_05228

296 chr3 5068857 5069357 ChIP_peak_180 MGG_05229

297 chr3 5100027 5100471 ChIP_peak_181 MGG_05239

298 chr3 5674304 5675044 ChIP_peak_183 MGG_16923

299 chr3 5674304 5675044 ChIP_peak_183 MGG_07765

300 chr3 5768250 5768621 ChIP_peak_184 MGG_07789

301 chr3 6121410 6121813 ChIP_peak_185 MGG_13891

302 chr3 6121410 6121813 ChIP_peak_185 MGG_16955

303 chr3 6121410 6121813 ChIP_peak_185 MGG_07876

304 chr3 6221688 6222162 ChIP_peak_186 MGG_13907

305 chr3 6221688 6222162 ChIP_peak_186 MGG_13908

306 chr3 6226667 6227327 ChIP_peak_187 MGG_07912

307 chr3 6226667 6227327 ChIP_peak_187 MGG_07910

308 chr3 6508781 6509296 ChIP_peak_188 MGG_10856

309 chr3 6508781 6509296 ChIP_peak_188 MGG_10855

310 chr3 6508781 6509296 ChIP_peak_188 MGG_10857

311 chr4 216841 217709 ChIP_peak_189 MGG_15347

312 chr4 216841 217709 ChIP_peak_189 MGG_08439

313 chr4 216841 217709 ChIP_peak_189 MGG_08440

314 chr4 452808 453397 ChIP_peak_191 MGG_08506

315 chr4 452808 453397 ChIP_peak_191 MGG_13974

316 chr4 549396 550152 ChIP_peak_192 MGG_13986

317 chr4 549396 550152 ChIP_peak_192 MGG_08535

318 chr4 553204 553596 ChIP_peak_193 MGG_08536

319 chr4 600389 601013 ChIP_peak_195 MGG_08547

320 chr4 600389 601013 ChIP_peak_195 MGG_17036

321 chr4 601752 602449 ChIP_peak_196 MGG_08547

322 chr4 601752 602449 ChIP_peak_196 MGG_17036

323 chr4 601752 602449 ChIP_peak_196 MGG_08548

324 chr4 892371 892687 ChIP_peak_198 MGG_17049

325 chr4 892371 892687 ChIP_peak_198 MGG_03731

326 chr4 892371 892687 ChIP_peak_198 MGG_15290

327 chr4 928747 929079 ChIP_peak_199 MGG_13474

328 chr4 928747 929079 ChIP_peak_199 MGG_15289

329 chr4 928747 929079 ChIP_peak_199 MGG_03716

330 chr4 1178540 1178905 ChIP_peak_201 MGG_03637

331 chr4 1178540 1178905 ChIP_peak_201 MGG_03636

332 chr4 1178540 1178905 ChIP_peak_201 MGG_13493

333 chr4 1178540 1178905 ChIP_peak_201 MGG_03635

334 chr4 1306839 1307338 ChIP_peak_202 MGG_03602

335 chr4 1467793 1468613 ChIP_peak_203 MGG_03559

336 chr4 1467793 1468613 ChIP_peak_203 MGG_03558

337 chr4 1558234 1558838 ChIP_peak_204 MGG_03536

338 chr4 1558234 1558838 ChIP_peak_204 MGG_03535

339 chr4 1558234 1558838 ChIP_peak_204 MGG_03537

340 chr4 1653933 1654318 ChIP_peak_205 MGG_03509

341 chr4 1653933 1654318 ChIP_peak_205 MGG_03508

342 chr4 1776530 1776964 ChIP_peak_207 MGG_03464

343 chr4 1776530 1776964 ChIP_peak_207 MGG_03463

344 chr4 1960053 1960476 ChIP_peak_208 MGG_03412

345 chr4 1960053 1960476 ChIP_peak_208 MGG_03411

346 chr4 2182674 2183629 ChIP_peak_210 MGG_03350

347 chr4 2182674 2183629 ChIP_peak_210 MGG_03349

348 chr4 2200478 2200920 ChIP_peak_211 MGG_03343

349 chr4 2200478 2200920 ChIP_peak_211 MGG_14879

350 chr4 2200478 2200920 ChIP_peak_211 MGG_03341

351 chr4 2217935 2218565 ChIP_peak_212 MGG_03337

352 chr4 2217935 2218565 ChIP_peak_212 MGG_03336

353 chr4 2217935 2218565 ChIP_peak_212 MGG_03335

354 chr4 2267890 2268200 ChIP_peak_213 MGG_03322

355 chr4 2267890 2268200 ChIP_peak_213 MGG_03321

356 chr4 2267890 2268200 ChIP_peak_213 MGG_03320

357 chr4 2332727 2333154 ChIP_peak_214 MGG_17118

358 chr4 2332727 2333154 ChIP_peak_214 MGG_03304

359 chr4 2375400 2375893 ChIP_peak_215 MGG_03291

360 chr4 2375400 2375893 ChIP_peak_215 MGG_17120

361 chr4 2375400 2375893 ChIP_peak_215 MGG_03290

362 chr4 2507512 2507832 ChIP_peak_216 MGG_14871

363 chr4 2507512 2507832 ChIP_peak_216 MGG_12742

364 chr4 2507512 2507832 ChIP_peak_216 MGG_03258

365 chr4 2698410 2698887 ChIP_peak_217 MGG_14868

366 chr4 2698410 2698887 ChIP_peak_217 MGG_03205

367 chr4 2871188 2871799 ChIP_peak_218 MGG_03153

368 chr4 2871188 2871799 ChIP_peak_218 MGG_12766

369 chr4 2871188 2871799 ChIP_peak_218 MGG_03154

370 chr4 2968727 2969085 ChIP_peak_219 MGG_17141

371 chr4 2968727 2969085 ChIP_peak_219 MGG_03131

372 chr4 3180625 3181157 ChIP_peak_221 MGG_17159

373 chr4 3180625 3181157 ChIP_peak_221 MGG_06590

374 chr4 3365640 3366512 ChIP_peak_222 MGG_17169

375 chr4 3365640 3366512 ChIP_peak_222 MGG_17170

376 chr4 3365640 3366512 ChIP_peak_222 MGG_06537

377 chr4 3456053 3456602 ChIP_peak_224 MGG_12816

378 chr4 3528721 3529214 ChIP_peak_225 MGG_12821

379 chr4 3553420 3553780 ChIP_peak_227 MGG_12822

380 chr4 3839696 3840214 ChIP_peak_231 MGG_06410

381 chr4 3839696 3840214 ChIP_peak_231 MGG_06409

382 chr4 4221462 4221808 ChIP_peak_232 MGG_06307

383 chr4 4221462 4221808 ChIP_peak_232 MGG_06306

384 chr4 4221462 4221808 ChIP_peak_232 MGG_06305

385 chr4 4619212 4619514 ChIP_peak_233 MGG_17222

386 chr4 4619212 4619514 ChIP_peak_233 MGG_17223

387 chr4 4619212 4619514 ChIP_peak_233 MGG_10106

388 chr4 4619212 4619514 ChIP_peak_233 MGG_10107

389 chr4 4800518 4800957 ChIP_peak_234 MGG_10050

390 chr4 4800518 4800957 ChIP_peak_234 MGG_10049

391 chr4 4851877 4852262 ChIP_peak_235 MGG_17241

392 chr4 4851877 4852262 ChIP_peak_235 MGG_10038

393 chr4 5019256 5019602 ChIP_peak_236 MGG_09995

394 chr4 5019256 5019602 ChIP_peak_236 MGG_09994

395 chr4 5062879 5063261 ChIP_peak_237 MGG_17255

396 chr4 5062879 5063261 ChIP_peak_237 MGG_09986

397 chr4 5062879 5063261 ChIP_peak_237 MGG_09985

398 chr4 5208854 5209249 ChIP_peak_238 MGG_09779

399 chr4 5208854 5209249 ChIP_peak_238 MGG_09778

400 chr4 5278850 5279266 ChIP_peak_239 MGG_09761

401 chr4 5358935 5359673 ChIP_peak_242 MGG_09734

402 chr4 5358935 5359673 ChIP_peak_242 MGG_13651

403 chr4 5362558 5363031 ChIP_peak_243 MGG_09734

404 chr4 5362558 5363031 ChIP_peak_243 MGG_09733

405 chr4 5427878 5428882 ChIP_peak_245 MGG_13654

406 chr4 5427878 5428882 ChIP_peak_245 MGG_13655

407 chr5 415183 415770 ChIP_peak_249 MGG_01127

408 chr5 415183 415770 ChIP_peak_249 MGG_11425

409 chr5 491034 491448 ChIP_peak_250 MGG_01108

410 chr5 491034 491448 ChIP_peak_250 MGG_01106

411 chr5 491034 491448 ChIP_peak_250 MGG_01107

412 chr5 528859 529239 ChIP_peak_251 MGG_01095

413 chr5 528859 529239 ChIP_peak_251 MGG_01094

414 chr5 624503 625227 ChIP_peak_254 MGG_01069

415 chr5 624503 625227 ChIP_peak_254 MGG_01068

416 chr5 688260 688769 ChIP_peak_255 MGG_01047

417 chr5 688260 688769 ChIP_peak_255 MGG_01046

418 chr5 848975 849383 ChIP_peak_257 MGG_01006

419 chr5 848975 849383 ChIP_peak_257 MGG_01005

420 chr5 848975 849383 ChIP_peak_257 MGG_01004

421 chr5 1077413 1078046 ChIP_peak_258 MGG_00933

422 chr5 1077413 1078046 ChIP_peak_258 MGG_00932

423 chr5 1350622 1351300 ChIP_peak_259 MGG_00861

424 chr5 1350622 1351300 ChIP_peak_259 MGG_00860

425 chr5 1350622 1351300 ChIP_peak_259 MGG_00859

426 chr5 1382541 1383231 ChIP_peak_261 MGG_17359

427 chr5 1382541 1383231 ChIP_peak_261 MGG_17360

428 chr5 1382541 1383231 ChIP_peak_261 MGG_17361

429 chr5 1709570 1710244 ChIP_peak_263 MGG_17371

430 chr5 1709570 1710244 ChIP_peak_263 MGG_17372

431 chr5 1834073 1834586 ChIP_peak_264 MGG_11502

432 chr5 1834073 1834586 ChIP_peak_264 MGG_00733

433 chr5 1898378 1898937 ChIP_peak_265 MGG_00716

434 chr5 1898378 1898937 ChIP_peak_265 MGG_00715

435 chr5 1898378 1898937 ChIP_peak_265 MGG_17389

436 chr5 2509522 2509847 ChIP_peak_268 MGG_17430

437 chr5 2509522 2509847 ChIP_peak_268 MGG_00549

438 chr5 2509522 2509847 ChIP_peak_268 MGG_00548

439 chr5 2595372 2595752 ChIP_peak_269 MGG_00529

440 chr5 2595372 2595752 ChIP_peak_269 MGG_00528

441 chr5 2960880 2961248 ChIP_peak_270 MGG_17460

442 chr5 3216677 3217240 ChIP_peak_271 MGG_14699

443 chr5 3216677 3217240 ChIP_peak_271 MGG_17471

444 chr5 3351126 3351805 ChIP_peak_273 MGG_00302

445 chr5 3351126 3351805 ChIP_peak_273 MGG_00301

446 chr5 3882925 3883434 ChIP_peak_275 MGG_00168

447 chr5 3882925 3883434 ChIP_peak_275 MGG_00167

448 chr5 3882925 3883434 ChIP_peak_275 MGG_00166

449 chr5 3929279 3929848 ChIP_peak_276 MGG_00153

450 chr5 3929279 3929848 ChIP_peak_276 MGG_00152

451 chr5 4046472 4047012 ChIP_peak_277 MGG_00127

452 chr5 4046472 4047012 ChIP_peak_277 MGG_00126

453 chr5 4147082 4147412 ChIP_peak_278 MGG_17537

454 chr5 4147082 4147412 ChIP_peak_278 MGG_00097

455 chr5 4147082 4147412 ChIP_peak_278 MGG_00096

456 chr5 4147082 4147412 ChIP_peak_278 MGG_00099

457 chr5 4186834 4187193 ChIP_peak_280 MGG_00082

458 chr5 4186834 4187193 ChIP_peak_280 MGG_00081

459 chr5 4186834 4187193 ChIP_peak_280 MGG_11676

460 chr5 4247670 4248248 ChIP_peak_282 MGG_00063

461 chr5 4247670 4248248 ChIP_peak_282 MGG_00065

462 chr5 4340110 4340488 ChIP_peak_283 MGG_00040

463 chr5 4340110 4340488 ChIP_peak_283 MGG_17546

464 chr5 4340110 4340488 ChIP_peak_283 MGG_00039

465 chr6 151111 151618 ChIP_peak_285 MGG_08776

466 chr6 376283 376711 ChIP_peak_287 MGG_08710

467 chr6 376283 376711 ChIP_peak_287 MGG_08709

468 chr6 800872 801207 ChIP_peak_288 MGG_12239

469 chr6 800872 801207 ChIP_peak_288 MGG_10131

470 chr6 1038602 1039217 ChIP_peak_289 MGG_10426

471 chr6 1038602 1039217 ChIP_peak_289 MGG_10427

472 chr6 1038602 1039217 ChIP_peak_289 MGG_10428

473 chr6 1194341 1195368 ChIP_peak_290 MGG_04321

474 chr6 1194341 1195368 ChIP_peak_290 MGG_17632

475 chr6 1194341 1195368 ChIP_peak_290 MGG_04319

476 chr6 1194341 1195368 ChIP_peak_290 MGG_04320

477 chr6 1480030 1480462 ChIP_peak_292 MGG_12184

478 chr6 1480030 1480462 ChIP_peak_292 MGG_04231

479 chr6 1521380 1521704 ChIP_peak_293 MGG_04217

480 chr6 1521380 1521704 ChIP_peak_293 MGG_17670

481 chr6 1543290 1543817 ChIP_peak_294 MGG_12176

482 chr6 1543290 1543817 ChIP_peak_294 MGG_04212

483 chr6 1711172 1711778 ChIP_peak_296 MGG_04176

484 chr6 1711172 1711778 ChIP_peak_296 MGG_04175

485 chr6 1711172 1711778 ChIP_peak_296 MGG_04174

486 chr6 1721773 1722430 ChIP_peak_298 MGG_17675

487 chr6 1721773 1722430 ChIP_peak_298 MGG_04171

488 chr6 1721773 1722430 ChIP_peak_298 MGG_04172

489 chr6 1772555 1772856 ChIP_peak_299 MGG_04159

490 chr6 1772555 1772856 ChIP_peak_299 MGG_04160

491 chr6 1945984 1946720 ChIP_peak_300 MGG_04106

492 chr6 1945984 1946720 ChIP_peak_300 MGG_04105

493 chr6 1945984 1946720 ChIP_peak_300 MGG_17686

494 chr6 1945984 1946720 ChIP_peak_300 MGG_04104

495 chr6 1945984 1946720 ChIP_peak_300 MGG_04103

496 chr6 2001713 2002659 ChIP_peak_301 MGG_04092

497 chr6 2001713 2002659 ChIP_peak_301 MGG_04091

498 chr6 2001713 2002659 ChIP_peak_301 MGG_04093

499 chr6 2041773 2042278 ChIP_peak_302 MGG_04077

500 chr6 2041773 2042278 ChIP_peak_302 MGG_04076

501 chr6 2041773 2042278 ChIP_peak_302 MGG_04075

502 chr6 2610627 2611096 ChIP_peak_306 MGG_03913

503 chr6 2610627 2611096 ChIP_peak_306 MGG_03912

504 chr6 3285085 3285796 ChIP_peak_307 MGG_09437

505 chr6 3285085 3285796 ChIP_peak_307 MGG_09436

506 chr6 3285085 3285796 ChIP_peak_307 MGG_09435

507 chr6 3361253 3361687 ChIP_peak_308 MGG_09414

508 chr6 3361253 3361687 ChIP_peak_308 MGG_09413

509 chr6 3361253 3361687 ChIP_peak_308 MGG_17785

510 chr6 3361253 3361687 ChIP_peak_308 MGG_09412

511 chr6 3505627 3506288 ChIP_peak_311 MGG_09373

512 chr6 3505627 3506288 ChIP_peak_311 MGG_09372

513 chr6 3600712 3601050 ChIP_peak_312 MGG_09350

514 chr6 3600712 3601050 ChIP_peak_312 MGG_09349

515 chr6 3600712 3601050 ChIP_peak_312 MGG_09348

516 chr6 3600712 3601050 ChIP_peak_312 MGG_09347

517 chr6 3660438 3660907 ChIP_peak_313 MGG_09330

518 chr6 3660438 3660907 ChIP_peak_313 MGG_09329

519 chr6 3719083 3719633 ChIP_peak_314 MGG_10724

520 chr6 3719083 3719633 ChIP_peak_314 MGG_10723

521 chr6 3719083 3719633 ChIP_peak_314 MGG_14930

522 chr6 4008618 4009244 ChIP_peak_316 MGG_15425

523 chr6 4008618 4009244 ChIP_peak_316 MGG_09821

524 chr6 4008618 4009244 ChIP_peak_316 MGG_09820

525 chr7 68033 68397 ChIP_peak_317 MGG_10334

526 chr7 68033 68397 ChIP_peak_317 MGG_10333

527 chr7 221248 221627 ChIP_peak_319 MGG_14202

528 chr7 221248 221627 ChIP_peak_319 MGG_10293

529 chr7 221248 221627 ChIP_peak_319 MGG_10292

530 chr7 221248 221627 ChIP_peak_319 MGG_15451

531 chr7 416736 417277 ChIP_peak_321 MGG_03066

532 chr7 416736 417277 ChIP_peak_321 MGG_03065

533 chr7 416736 417277 ChIP_peak_321 MGG_03064

534 chr7 538356 539017 ChIP_peak_323 MGG_03042

535 chr7 538356 539017 ChIP_peak_323 MGG_17880

536 chr7 538356 539017 ChIP_peak_323 MGG_03041

537 chr7 671503 672102 ChIP_peak_325 MGG_03008

538 chr7 671503 672102 ChIP_peak_325 MGG_03007

539 chr7 763930 764445 ChIP_peak_326 MGG_17900

540 chr7 763930 764445 ChIP_peak_326 MGG_17901

541 chr7 763930 764445 ChIP_peak_326 MGG_17902

542 chr7 859661 859993 ChIP_peak_328 MGG_17908

543 chr7 859661 859993 ChIP_peak_328 MGG_17909

544 chr7 897918 898308 ChIP_peak_329 MGG_02946

545 chr7 1119161 1119880 ChIP_peak_331 MGG_02893

546 chr7 1119161 1119880 ChIP_peak_331 MGG_17939

547 chr7 1266566 1266869 ChIP_peak_332 MGG_02854

548 chr7 1266566 1266869 ChIP_peak_332 MGG_02853

549 chr7 1451681 1452516 ChIP_peak_333 MGG_02806

550 chr7 1451681 1452516 ChIP_peak_333 MGG_02805

551 chr7 1451681 1452516 ChIP_peak_333 MGG_02804

552 chr7 1456865 1457351 ChIP_peak_334 MGG_02804

553 chr7 1456865 1457351 ChIP_peak_334 MGG_02803

554 chr7 1456865 1457351 ChIP_peak_334 MGG_02802

555 chr7 1808149 1808598 ChIP_peak_338 MGG_02704

556 chr7 1808149 1808598 ChIP_peak_338 MGG_15015

557 chr7 1808149 1808598 ChIP_peak_338 MGG_02702

558 chr7 1808149 1808598 ChIP_peak_338 MGG_02705

559 chr7 1989069 1989951 ChIP_peak_341 MGG_02665

560 chr7 1989069 1989951 ChIP_peak_341 MGG_12627

561 chr7 1989069 1989951 ChIP_peak_341 MGG_12628

562 chr7 2168571 2169131 ChIP_peak_342 MGG_12647

563 chr7 2168571 2169131 ChIP_peak_342 MGG_02623

564 chr7 2168571 2169131 ChIP_peak_342 MGG_02622

565 chr7 2218556 2218891 ChIP_peak_343 MGG_02607

566 chr7 2218556 2218891 ChIP_peak_343 MGG_02606

567 chr7 2218556 2218891 ChIP_peak_343 MGG_02605

568 chr7 2218556 2218891 ChIP_peak_343 MGG_02604

569 chr7 2251410 2251948 ChIP_peak_346 MGG_18018

570 chr7 2446633 2446988 ChIP_peak_347 MGG_02538

571 chr7 2446633 2446988 ChIP_peak_347 MGG_12670

572 chr7 2446633 2446988 ChIP_peak_347 MGG_12671

573 chr7 2877981 2878309 ChIP_peak_349 MGG_10562

574 chr7 3261384 3261769 ChIP_peak_350 MGG_09602
